# Altered Mental Status Secondary to Extensive Pneumocephalus

**DOI:** 10.5811/cpcem.2017.6.34503

**Published:** 2017-10-03

**Authors:** Alexander J. Scumpia, Gary Lai, Maria I. Rodriguez, Daniel M. Aronovich, Gregory Dubrovich, Sopiko Jimsheleishvili

**Affiliations:** * Broward Health, Imperial Point Hospital, Department of Emergency Medicine, Fort Lauderdale, Florida; † Broward Health, Coral Springs Hospital, Department of Emergency Medicine, Coral Springs, Florida; ‡ University of California San Francisco, Department of Global Health Sciences, San Francisco, California

## CASE PRESENTATION

A 58-year-old-male Caucasian presented to the emergency department (ED) with altered mental status and progressively worsening generalized weakness for three days, status-post endoscopic sinus surgery. The patient’s family described “clear liquid” leaking from the patient’s right nostril earlier in the day. On physical exam the patient was hemodynamically stable, airway patent, alert only to person, and moving all extremities. Epistaxis or rhinorrhea was not observed in the ED. Plain computed tomography (CT) of the brain demonstrated extensive pneumocephalus with five centimeters of bifrontal extra-axial gas and a small subdural hematoma along the inferior-anterior margin of the interhemispheric fissure adjacent to the gyrus rectus (Image). Following immediate neurosurgical consultation, we initiated conservative management that included bed rest, head-of-bed elevation to 35 degrees and intravenous (IV) fluid administration. Further observation and CT cisternography failed to demonstrate a cerebrospinal fluid leak or fistula formation. The patient made an uneventful recovery and was subsequently discharged home three days later.

## DISCUSSION

Pneumocephalus has been defined as the presence of intracranial gas.^1^ Clinically, it is essential that the emergency physician distinguish tension pneumocephalus (gas under pressure) from simple pneumocephalus (i.e., pneumocephalus from a craniotomy). Gas may be present in the epidural, subdural, subarachnoid (as was the case described herein), intraparenchymal and or intraventricular space.^1^ The most common presentations of pneumocephalus are headaches, nausea and/or vomiting, dizziness and obtundation.^2^

Tension pneumocephalus encompasses signs of increased intracranial pressure such as a focal deficit from mass effect of an intracranial lesion. Pneumocephalus can be caused by craniotomy, ventriculoperitoneal shunt placement, subdural hematoma evacuation via burr-hole(s), post-traumatic (basal skull) fracture, or iatrogenic effect following sinus surgery, as was the case here.^3^ Furthermore, gas-producing organisms (i.e. infection), lumbar puncture, spinal anesthesia, and barotrauma and can be culprits as well. The diagnosis is easily accomplished via CT.^1^

Most cases of pneumocephalus do not require neurosurgical intervention; however, tension pneumocephalus may require surgery. Surgical treatment may range from additional burr-holes or insertion of spinal needles through already existing burr-holes.^1^ Gas-producing organisms need to be treated accordingly and pneumocephalus secondarily.^3^ Non-infectious, simple pneumocephalus is treated conservatively via head elevation, IV fluids, bed rest and neuro checks. If a cerebrospinal fluid leak or fistula is suspected, CT cisternography can be used.^1^ Although uncommon, simple pneumocephalus vs. tension pneumocephalus must be diagnosed quickly along with early neurosurgical consultation. This case illustrates the broad differential diagnosis that the emergency physician must contemplate when working up a patient with altered mental status.

**CPC-EM Capsule**

What do we already know about this clinical entity?

Pneumocephalus has been defined as intracranial gas usually following craniotomies and rarely endoscopic sinus surgeries, as described herein.

What is the major impact of the image(s)?

The image illustrates multiple intracranial pathologies including: extensive pneumocephalus, intraventricular gas, subarachnoid hemorrhage and subdural gas with midline shift.

How might this improve emergency medicine practice?

Altered mental status has many etiologies, pneumocephalus should not be overlooked. Imaging, along with early specialist consultation is essential in decreasing morbidity and mortality for our patients.

## Figures and Tables

**Image:**
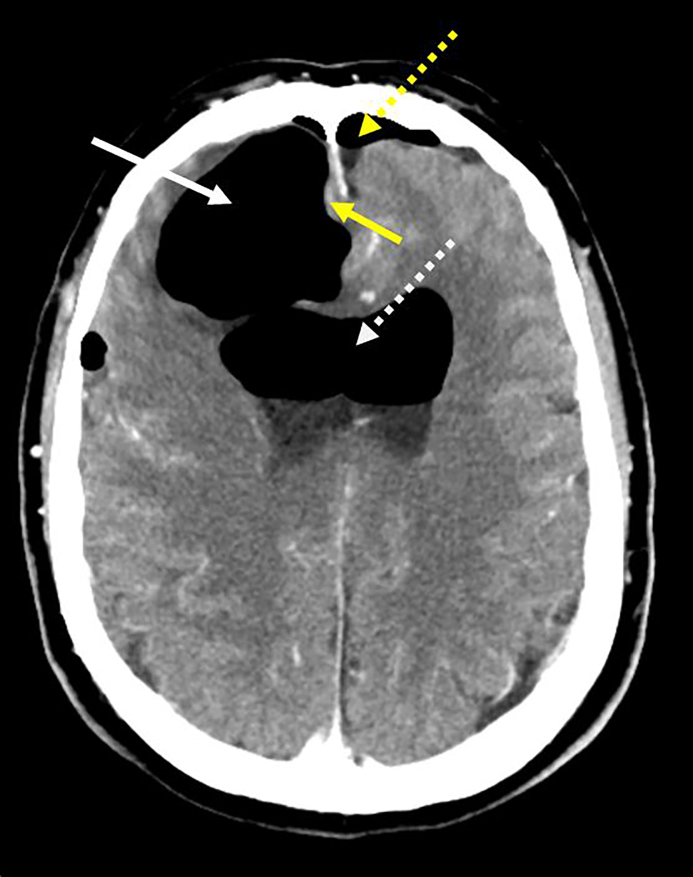
Computed tomography (axial view) of the brain demonstrating extensive, right greater than left, frontal pneumocephalus (solid white arrow), subarachnoid (yellow dotted arrow) and bilateral frontal ventricular horn gas (white dotted arrow) without midline shift. The CT also demonstrates minimal subdural blood (yellow arrow) along the inferior-anterior margin of the interhemispheric fissure adjacent to the gyrus rectus.
